# Menstrual health outcomes and associations with depression and anxiety in Spain: a cross-sectional study

**DOI:** 10.1080/26410397.2026.2702119

**Published:** 2026-07-31

**Authors:** Andrea García-Egea, Tomàs López-Jiménez, Uxune Apalategi-Gómez, Maria José Fernández-Domínguez, Carmen Rodríguez-Domínguez, Vinita Mahtani-Chugani, Anna Berenguera, Laura Medina-Perucha

**Affiliations:** a Research Technician, Fundació Institut Universitari per a la recerca a l'Atenció Primària de Salut Jordi Gol i Gurina (IDIAPJGol), Barcelona, Spain; Universitat Autònoma de Barcelona, Bellaterra (Cerdanyola del Valles), Spain; b Research Technician, Fundació Institut Universitari per a la recerca a l'Atenció Primària de Salut Jordi Gol i Gurina (IDIAPJGol), Barcelona, Spain; Universitat Autònoma de Barcelona, Bellaterra (Cerdanyola del Vallès), Spain; c Research Technician, Network for Research on Chronicity, Primary Care and Health Promotion (RICAPPS), Spain; Osakidetza-Servicio Vasco de Salud, Organización Sanitaria Integrada Donostialdea, Unidad de Epidemiología Clínica e Investigación, Donostia-San Sebastián, Spain; Osakidetza-Servicio Vasco de Salud, Instituto de Investigación Sanitaria Biogipuzkoa, Donostia-San Sebastián, Spain; d Researcher, Network for Research on Chronicity, Primary Care and Health Promotion (RICAPPS), Spain; Centro de Saúde Leiro, Área sanitaria de Ourense, Spain; Gerencia de Atención Primaria de Tenerife, Servicio Canario de Salud, Santa Cruz de Tenerife, Spain; e Associate Professor, Departamento de Psicología Social, Evolutiva y de la Educación, Universidad de Huelva, Huelva, Spain; f Researcher, Network for Research on Chronicity, Primary Care and Health Promotion (RICAPPS), Spain; Unidad de Investigación Hospital Universitario Nuestra Señora de Candelaria, Spain; Grupo I-Saúde, Instituto de Investigación Sanitaria Galicia Sur, Vigo, Spain; L'Associació de Drets Sexuals i Reproductius, Barcelona, Spain; Grupo de Investigación en Salut Sexual y Reproductiva (GRASSIR), Barcelona, Spain; Atención a la Salud Sexual y Reproductiva (ASSIR), Dirección asistencial de Atención Primaria, Institut Català de la Salut, Barcelona, Spain; Facultad de Enfermeria, Universidad de Barcelona, Spain; Osakidetza-Servicio Vasco de Salud, Subdirección de Asistencia Sanitaria, Unidad Docente Multiprofesional de Atención Familiar y Comunitaria de Gipuzkoa, Donostia-San Sebastián, Spain; Network for Research on Chronicity, Primary Care and Health Promotion (RICAPPS), Spain; Grupo I-Saúde, Instituto de Investigación Sanitaria Galicia Sur, Vigo, Spain; Departamento de Psicología Experimental, Universidad de Sevilla, Sevilla, Spain; Gerencia de Atención Primaria de Tenerife, Servicio Canario de Salud, Santa Cruz de Tenerife, Spain; Network for Research on Chronicity, Primary Care and Health Promotion (RICAPPS), Spain; Departamento de Psicología Experimental, Universidad de Sevilla, Sevilla, Spain; Osakidetza-Servicio Vasco de Salud, Subdirección de Asistencia Sanitaria, Unidad de Investigación de Atención Primaria y OSIs de Gipuzkoa, Donostia-San Sebastián, Spain; Network for Research on Chronicity, Primary Care and Health Promotion (RICAPPS), Spain; Osakidetza-Servicio Vasco de Salud, Subdirección de Asistencia Sanitaria, Unidad de Investigación de Atención Primaria y OSIs de Gipuzkoa, Donostia-San Sebastián, Spain; Centro de Saúde A Milagrosa. Área Sanitaria de Lugo, Spain; Departamento de Psicología, Universidad Loyola Andalucía, Dos Hermanas, Seville, Spain; Centro de Análisis Y Programas Sanitarios (CAPS), Barcelona, Spain; Area sanitaria de Ourense, Verin e Barco Valdeorras, Spain; Instituto de Investigación Sanitaria Galicia Sur, Vigo, Spain; Facultad de ciencias de la educación y del trabajo social, Universidad de Vigo, Spain; Departamento de Obstetricia y Ginecología, Hospital Universitario de Valme, Sevilla, Spain; Departamento de Psicología Evolutiva y de la Educación, Instituto de Biomedicina de Sevilla (IBIS), Universidad de Sevilla, Sevilla, Spain; g Senior Researcher, Fundació Institut Universitari per a la recerca a l'Atenció Primària de Salut Jordi Gol i Gurina (IDIAPJGol), Barcelona, Spain; Universitat Autònoma de Barcelona, Bellaterra (Cerdanyola del Vallès), Spain; Network for Research on Chronicity, Primary Care and Health Promotion (RICAPPS), Spain; Departament d’Infermeria, Universitat de Girona, Girona, Spain; h Senior Researcher, Fundació Institut Universitari per a la recerca a l'Atenció Primària de Salut Jordi Gol i Gurina (IDIAPJGol) Barcelona, Spain; Universitat Autonoma de Barcelona, Bellaterra (Cerdanyola del Valles), Spain; Network for Research on Chronicity, Primary Care and Health Promotion (RICAPPS), Spain

**Keywords:** menstruation, menstrual health, depression, anxiety, social inequities of health, women’s health

## Abstract

This study analysed the relationship between menstrual health and self-reported depression and anxiety in women and people who menstruate (PWM) (≥18 years) in Spain, from a critical gender perspective. An online survey was used to collect data in 32 healthcare centres in 2023. Descriptive and ordinal logistic regression models were performed with 1,404 participants. Menstrual bleeding of over 80 ml was associated with higher odds of more severe depression (aOR = 1.61; 95% CI: 1.45–1.79; *p* < 0.001) and anxiety (aOR = 1.67; 95% CI: 1.35–2.06; *p* < 0.001). Higher menstrual pain intensity was also linked to higher odds of more severe depression (aOR =  2.05; 95% CI: 1.82–2.31; *p* < 0.001) and anxiety (aOR = 1.82; 95% CI: 1.43–2.30; *p* < 0.001). A higher frequency of premenstrual symptoms was associated with increased odds for more severe depression (aOR = 14.54; 95% CI: 10.73–19.70; *p* < 0.001) and anxiety (aOR = 12.95; 95% CI: 9.10–18.44; *p* < 0.001). In addition, higher reports of menstrual taboo, stigma and discrimination were associated with higher odds of more severe depression (aOR = 2.23; 95% CI: 1.81–2.76; *p* < 0.001) and anxiety (aOR = 2.34; 95% CI: 1.91–2.87; *p* < 0.001). Reports of more severe depression (aOR = 2.50; 95% CI: 1.65–3.78; *p* < 0.001) and anxiety (aOR =  2.55; 95% CI: 1.70–3.83; *p* < 0.001) increased with the severity of menstrual poverty. Greater impact on social participation during menstruation was strongly associated with higher odds of more severe depression (aOR = 7.41; 95% CI: 4.90–11.191; *p* < 0.001) and anxiety (aOR = 6.69; 95% CI: 5.22–8.59; *p* < 0.001). Sociodemographic factors may mediate the menstrual–mental health relationship. Our study sheds light on the interplay between menstrual and mental health in our context, from a gender and social health inequities perspective.

## Plain language summary

There is little research on how menstrual health is linked to other health outcomes, especially with emotional and mental health. This study analysed whether menstrual health is associated with depression and anxiety in women and people who menstruate (PWM) aged 18 years or older. This study was conducted in 32 healthcare centres across five Spanish regions (Andalusia, Basque Country, Canary Islands, Catalonia and Galicia). We developed an online survey and collected data during 2023. The data showed depression and anxiety were more likely in the following cases: (1) when there was heavy menstrual bleeding, (2) when there was intense menstrual pain, (3) where there were high levels of menstrual stigma, taboo or discrimination, (4) when women and PWM frequently lacked access to menstrual products and (5) when menstruation made it harder for women and PWM to take part in social activities. Other factors (e.g. age, financial hardship) also seemed to affect menstrual health and depression or anxiety. To sum up, this study shows a connection between poorer menstrual health and depression and anxiety.

## Résumé

Cette étude a analysé, dans une perspective critique de genre, la relation entre la santé menstruelle et la dépression et l’anxiété autodéclarées chez les femmes et les personnes menstruées (âgées de plus de 18 ans) en Espagne. Une enquête en ligne a permis de recueillir des données dans 32 centres de santé en 2023. Des modèles de régression logistique descriptive et ordinale ont été réalisés auprès de 1404 participantes. Des saignements menstruels supérieurs à 80 ml étaient associés à une probabilité accrue de dépression (RCa = 1.61; IC 95%: 1.45–1.79; *p* < 0.001) et d’anxiété (RCa = 1.67; IC 95%: 1.35–2.06; *p* < 0.001) plus sévères. Une intensité plus élevée des douleurs menstruelles était aussi associée à une probabilité accrue de dépression (RCa = 2.05; IC 95%: 1.82–2.31; *p* < 0.001) et d’anxiété (RCa = 1.82; IC 95%: 1.43–2.30; *p* < 0.001) plus sévères. Une fréquence plus élevée de symptômes prémenstruels était associée à une probabilité accrue de dépression (RCa = 14.54; IC 95%: 10.73–19.70; *p* < 0.001) et d’anxiété (RCa = 12.95; IC 95%: 9.10–18.44; *p* < 0.001) plus sévères. De plus, une plus grande fréquence de tabous, de stigmatisation et de discrimination en lien avec les menstruations était associée à une probabilité accrue de dépression (RCa = 2.23; IC 95%: 1.81–2.76; *p* < 0.001) et d’anxiété (RCa = 2.34; IC 95%: 1.91–2.87; *p* < 0.001) plus sévères. La fréquence des cas de dépression (RCa = 2.50; IC 95%: 1.65–3.78; *p* < 0.001) et d’anxiété (RCa = 2.55; IC 95%: 1.70–3.83; *p* < 0.001) plus sévères augmentait avec la gravité de la précarité menstruelle. Un impact plus important sur la participation sociale pendant les menstruations était fortement associé à une probabilité accrue de dépression (RCa = 7.41; IC 95%: 4.90–11,191; *p* < 0.001) et d’anxiété (RCa = 6.69; IC 95%: 5.22–8.59; *p* < 0.001) plus sévères. Les facteurs sociodémographiques peuvent régir les relations entre santé menstruelle et santé mentale. Notre étude met en lumière les interactions entre santé menstruelle et santé mentale dans notre contexte, du point de vue des inégalités liées au genre et à la santé sociale.

## Resumen

Este estudio analizó la relación entre la salud menstrual y la depresión y ansiedad autoinformadas por mujeres y personas que menstrúan (PWM) (≥18 años) en España, desde una perspectiva de género crítica. En 2023, se utilizó una encuesta digital para recolectar datos en 32 centros de salud. Se aplicaron modelos descriptivos y de regresión logística ordinal con 1404 participantes. El sangrado menstrual superior a 80 ml se relacionó con mayores probabilidades de depresión más grave (RMa = 1.61; IC del 95%: 1.45–1.79; *p* < 0.001) y ansiedad (RMa = 1.67; IC del 95%: 1.35–2.06; *p* < 0.001). Una mayor intensidad de dolor menstrual también se relacionó con mayores probabilidades de depresión más severa (RMa = 2.05; IC del 95%: 1.82–2.31; *p* < 0.001) y ansiedad (RMa = 1.82; IC del 95%: 1.43–2.30; *p* < 0.001). Una mayor frecuencia de síntomas premenstruales se relacionó con mayores probabilidades de depresión más grave (RMa = 14.54; IC del 95%: 10.73–19.70; *p* < 0.001) y ansiedad (RMa = 12.95; IC del 95%: 9.10–18.44; *p* < 0.001). Además, un mayor número de informes de tabú, estigma y discriminación menstruales se relacionaron con mayores probabilidades de depresión más grave (RMa = 2.23; IC del 95%: 1.81–2.76; *p* < 0.001) y ansiedad (RMa = 2.34; IC del 95%: 1.91–2.87; *p* < 0.001). Los informes de depresión más grave (RMa = 2.50; IC del 95%: 1.65–3.78; *p* < 0.001) y ansiedad (RMa = 2.55; IC del 95%: 1.70–3.83; *p* < 0.001) aumentaron con la gravedad de la pobreza menstrual. Un mayor impacto en la participación social durante la menstruación se relacionó marcadamente con mayores probabilidades de depresión más grave (RMa = 7.41; IC del 95%: 4.90–11.191; *p* < 0.001) y ansiedad (RMa = 6.69; IC del 95%: 5.22–8.59; *p* < 0.001). Los factores sociodemográficos podrían mediar la relación entre la menstruación y la salud mental. Nuestro estudio arroja luz sobre la interrelación entre la menstruación y la salud mental en nuestro contexto, desde una perspectiva de género e inequidades de salud social.

## Introduction

Research on equity and menstrual health has increased, especially in the last decade, driven by feminist social movements advocating for greater visibility of menstrual experiences of women and people who menstruate (PWM).^1^ The field of menstrual research has proposed definitions of concepts such as *menstrual health*, understood as “*a complete state of physical, mental and social well-being and not merely the absence of disease, in relation to the menstrual cycle*”.^2^ This definition indicates that menstrual health should be ensured through: (1) adequate access to information about the menstrual cycle and menstruation, as well as self-care and hygiene practices; (2) access to products, spaces and services for menstrual management; (3) access to early diagnosis and treatment of health conditions related to the menstrual cycle; (4) being able to experience menstruation without stigma, discomfort or psychological distress; and (5) being able to decide how to participate in different spheres of life, including civil, cultural, economic, social and political spheres throughout the menstrual cycle.^2^ The development of this research field involves reviewing and suggesting new ways of conceptualising and operationalising menstrual health. These processes should consider the narratives of women and PWM, analyse the institutionalisation of menstrual discourses, and promote the collective construction of meanings and conceptual models.^3,4^

Available evidence suggests a correlation between menstrual health and physical health,^5^ mental and emotional health,^6,7^ and social and community participation.^7,8^ Consequently, menstrual health ought to be considered in the context of its interrelationship with other health concerns and other domains of life. Previous literature has demonstrated an association between menstrual health-related domains (e.g. premenstrual syndrome, menstrual poverty) and emotional and mental health outcomes, especially depression and anxiety,^6,9–11^ as well as the exacerbation of mental health disorders.^12^ However, the relationship between menstrual health^2^ and other health outcomes remains understudied in the Spanish context. The integration of menstrual health and mental health should be treated as a priority. Research teams should take a lead in contributing to the development of alternative menstrual discourses that explore the emotional and mental health experiences of women and PWM. From the perspectives of public health, gender and feminist health research, this involves a critical analysis of the context in which women and PWM menstruate^7,8,13^ and, in particular, the specific contexts within which women and PWM navigate their emotional and mental health. Moreover, it signifies the necessity to deliberate on the manner in which mental health conceptualisations often pathologise women's discomfort, thereby overlooking the structural factors* (e.g. gender norms, socioeconomic inequities, etc.) that contribute to this discomfort.^14,15^ It is imperative to acknowledge that the psychiatrisation of women and PWM is not limited to the menstrual experience, but frequently encompasses other embodied experiences, especially those associated with pain.^14,15^ The concept of psychiatrisation involves framing emotional discomfort through self-policing and self-corrective processes (e.g. judging one’s emotional and pain experiences, or enforcing hegemonic gender roles on oneself),^16^ as well as through biomedicalisation.†^17^

Primary health care is the first point of contact and a key provider of services for promoting menstrual health^7,8^ and delivering mental health consultations in Spain.^18^ In the wake of the COVID-19 mental health crisis, strategies for improving mental health in Spain have been strengthened, particularly in relation to depression and anxiety. The Mental Health Strategy 2022–2026 focuses on prevention, early detection, effective care and a comprehensive and community-based approach. Its overarching goal is to strengthen the national health system’s capacity to address mental health, grounded in equity and an integrative approach to health.^18^ This perspective underscores the need to examine how mental health intersects with different areas of life, including the menstrual realities of women and PWM.

This study proposes the following hypotheses: (1) there is an association between menstrual health and mental health, whereby menstrual health problems are associated with poorer mental health outcomes (especially anxiety and depression) and (2) this association varies according to socio-demographic characteristics. Accordingly, this study aimed to analyse the relationship between menstrual health, considering its integral definition informed by Hennegan et al 2021,^2^ and self-reported experiences of depression and anxiety in women and PWM (≥18 years) across five regions of Spain (Andalusia, Basque Country, Canary Islands, Catalonia and Galicia).

## Methods

A multicentric cross-sectional study was conducted, applying a critical gender perspective and grounded in health equity principles. A critical gender perspective examines how gender norms and power structures shape health processes. This approach challenges the androcentrism,‡ which plays a significant role in rendering menstrual health invisible.^19^ Moreover, a health equity perspective emphasises the importance of reducing health disparities and ensuring individuals’ right to health and well-being. Applying an equity lens is particularly relevant in menstrual health research, as menstruation is experienced differently across population groups.

The study was conducted in 32 healthcare centres between May and September 2023 across five regions in Spain: Andalusia (*N* = 7), Basque Country (*N* = 6), Canary Islands (*N* = 6), Catalonia (*N* = 7) and Galicia (*N* = 6). These regions represent approximately 48% (*N* = 23,318,351) of the total population in Spain and 48% (*N* = 11,877,315) of women living in Spain, according to the Instituto Nacional de Estadística (www.ine.es) (see Figure 1). The STROBE guidelines^20^ were followed to ensure transparent and comprehensive reporting of this observational study (see Supplementary Material 1).

**Figure 1.**
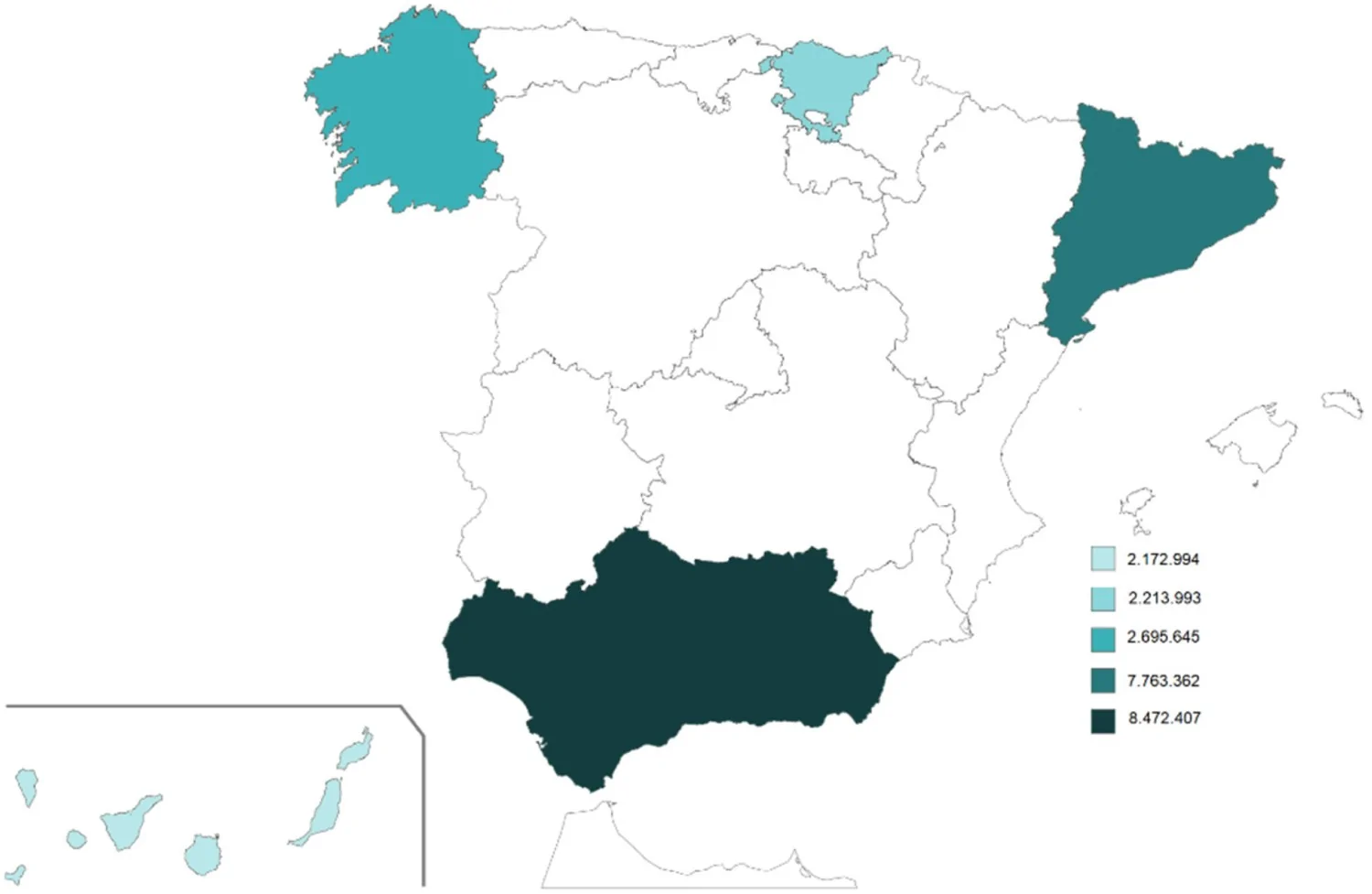
Graphic representation of participating regions, by population count. Retrieved from Instituto Nacional de Estadística: https://www.ine.es/jaxiT3/Tabla.htm?t=2853

### Sampling, recruitment and data collection

Participants met the following inclusion criteria: (1) women and PWM with active menstrual cycles (at least one menstruation in the last 6 months); (2) aged 18 or over; (3) living in Andalusia, Basque Country, Canary Islands, Catalonia, and Galicia; (4) users of a participating healthcare centre. A minimum of 1,141 women and PWM was required (229 participants/region). The sample size was calculated using a conventional binary logistic regression, assuming an odds ratio (OR) of 1.5 for the association between a given exposure and the presence (vs. absence) of depressive or anxiety symptoms, assuming 80% statistical power, a 5% alpha risk, and an interclass correlation (ICC) of 0.01 between healthcare centres. This binary approach was adopted as a conservative strategy, as the primary analyses were conducted using ordinal logistic regression models that make use of the full ordered outcome information. This effect size (OR = 1.5) represents a moderate and clinically relevant difference in presenting worse mental health outcomes, consistent with recommendations for population-based research.^21^ The ICC of 0.01 accounts for the potential similarity between participants within the same healthcare centre and aligns with standard assumptions for primary health care research.^22^ The final required sample was distributed equally across the five selected regions (*n* = 229 per region), ensuring geographic representativeness and logistical feasibility. This calculation approach follows established survey research methods.^23^ Based on our minimal sample size (*n* = 1,141) and assuming a balanced distribution of outcomes and exposures, the study had sufficient power to detect odds ratios of 1.5 or greater with at least 80% statistical power. Sample size calculations were performed using PASS software [PASS 2022 Power Analysis and Sample Size Software. (2022) NCSS, LLC. Kaysville, Utah, USA].

Sampling was non-probabilistic, combining convenience and purposive strategies. Convenience sampling facilitated participant recruitment based on availability and accessibility during data collection. Purposive sampling was employed to ensure inclusion of individuals with specific characteristics aligned with the research aim. Participants were recruited from six primary healthcare and sexual and reproductive healthcare centres in each region, except for Catalonia and Andalusia, which each included seven centres. In total, 32 centres participated in the study. The research team identified participating centres to ensure diversity in socioeconomic characteristics of the population in each healthcare centre. Once the centres agreed to participate, healthcare professionals were invited to recruit participants. Recruiters were provided with a guide outlining all necessary information for the recruitment process. The study sought to ensure diversity in age (18–25; 26–35; 36–45; >46), migration status (born in/outside Spain), inclusion of participants from the LGTBQ+ community and people with disabilities. The research team monitored the recruitment process, providing recruiters with fortnightly updates on the profiles of participants already recruited and those still required at each centre. This allowed adjustments to recruitment efforts to ensure the inclusion of participants with the desired characteristics.

A recruitment poster and a participant information sheet were used to generate interest and encourage participation in the study. Once women and PWM agreed to participate, healthcare professionals provided them with access to the data collection questionnaire, hosted on the Limesurvey platform. This is a secure, web-based software platform designed for conducting online surveys (https://www.limesurvey.org). Participants self-completed the questionnaire at the healthcare centre, in approximately 20 minutes. A paper version of the questionnaire was offered to participants who were unable to complete it online. The research team provided biweekly updates to each healthcare centre on the progress of recruitment and data collection.

The questionnaire was developed by the research team, an interdisciplinary group of experts (e.g. psychologists, physicians, midwives, statisticians). Validated instruments were used for some variables, while other items were specifically designed by the researchers. Four meetings were held to design the questionnaire, which was subsequently piloted in all regions prior to data collection. Although the minimum required sample size was 1,141, a total of 2,153 questionnaires were collected. This oversampling ensured adequate representation across different sociodemographic characteristics, facilitating stratified analysis. It also allowed for potential exclusions due to non-eligibility or missing data. Of the 2,153 questionnaires, 1,431 participants met the study’s inclusion criteria (age ≥18 years, having had at least one period in the last 6 months). Among these, 1,404 participants had complete data for all relevant variables and were included in the final analysis. Given the low proportion of missing data (1.4%), complete case analysis was performed, an approach considered methodologically acceptable under such conditions.^24^

### Variables

Data on sociodemographic characteristics, menstrual health and mental health were collected. Sociodemographic variables were: age, region of residence in Spain, gender identity, sexual orientation, country of birth, administrative status, level of education completed, employment status, possession of a public healthcare card, financial constraints, informal caregiving, experiences of discrimination and recognised disability.

Data on menstrual health were collected primarily through questions on: age at menarche, menstrual bleeding abundance,§ presence of menstrual blood clots, menstrual bleeding duration, menstrual cycle duration, intensity of menstrual pain, self-reported gynaecological and systemic health conditions,** use and type of hormonal contraception, frequency of premenstrual symptoms, impact of premenstrual symptoms on daily life, comfort in changing menstrual products at home, at work and in public spaces, access to menstrual education, menstrual-related experiences of taboo, stigma and discrimination, satisfaction with menstrual healthcare consultations, menstrual poverty and impairment of social participation during menstruation. The questions used to collect these variables were developed by the research team, drawing on their expertise in the menstrual health field and the prior use of some of these items in previous studies.^8^ The variable “social participation due to menstruation” was constructed from items of the validated CVM-22 scale.^25^ These items included information on work, domestic and academic activities during menstruation.†† Although the initial intention was to use the full scale, the research team decided to employ only selected items to avoid redundancy with other scales already included in the study.

Premenstrual symptoms were assessed using a 12-item question addressing emotional fluctuations (e.g. sadness) and physical changes (e.g. tiredness) experienced in the week or two weeks prior to menstrual bleeding. Access to menstrual education was evaluated using a two-item question. Experiences of menstrual-related taboo, stigma and discrimination were assessed through a four-item question. Menstrual poverty was measured using a three-item question that explored: (1) financial difficulties in purchasing menstrual products in the six months prior to the study; (2) financial difficulties in affording preferred menstrual products during the same period and (3) prolonged use of menstrual products beyond the recommended duration to save money in the six months prior to the study. Social participation was assessed using responses to 9 items from the CVM-22 scale, focusing on reduced productivity during menstruation, menstruation-related work absenteeism and declining social plans while menstruating, along with one additional item addressing the impact of premenstrual symptoms on daily life. Due to an unintentional omission during the adaptation of the questionnaire to the LimeSurvey platform, three CVM-22 items used to construct the social participation variable were not included in the first 591 online questionnaires. Analyses related to the social participation variable were conducted excluding these three items. Items were answered using a six-point Likert-type scale, except for social participation, which was measured using a four-point Likert-type scale from a validated questionnaire.

Mental health variables were assessed using two validated scales: the Patient Health Questionnaire-9 (PHQ-9) for depressive symptoms,^26^ and the General Anxiety Disorder-7 (GAD-7) scale for anxiety symptoms.^27^ In addition, a non-validated question was developed to collect self-reported mental health diagnoses. A validated single-item measure of self-rated health was also included (“In general, how would you say your health is?”), with responses categorised on a five-point Likert scale (excellent; very good; good; fair; poor).

Depression and anxiety were coded as ordered categorical outcomes. Depression (PHQ-9) was categorised into five ordered levels (none, mild, moderate, moderately severe, severe) and anxiety (GAD-7) into four ordered levels, following standard cut-offs. Both outcomes were analysed using proportional odds ordinal logistic regression models. The variables on the impact of premenstrual symptoms, access to menstrual education, menstruation-related experiences of taboo, stigma and discrimination, menstrual poverty and social participation were constructed using complex algorithm categorisations. These categorisations were defined during the initial stages of the analytical process. For the frequency of premenstrual symptoms (12-items question), each item had six possible response options ranging from “never” to “always”, scored from 0 to 5. A total score was calculated by summing the responses, resulting in a possible range of 0–60. This variable was then recoded into three categories: low (0–20), moderate (21–40) and high (41–60) frequency of premenstrual symptoms. The same procedure was applied to the following variables: menstrual poverty [never (0); low (1–3), moderate (4–6), high (7–15)]; access to menstrual education [low (0–3), moderate (4–6) and high (7–10)]; menstruation-related experiences of taboo, stigma, and discrimination [low (0–3), moderate (4–6) and high (7–20)]; and social participation [mildly affected (0–8), moderately affected (9–16), significantly affected (17–24) and highly affected (25–33)].

### Data analysis

Descriptive statistics were calculated for all variables to assess asymmetric distributions. Categorical variables were reported as frequencies (percentages), while continuous variables were presented as means with standard deviations (SD). Group differences were assessed using Chi-square and ANOVA tests. A separate statistical model was estimated for each menstrual health exposure of interest, resulting in 15 models for depression and 15 models for anxiety. In each model, the exposure of interest was entered as the main independent variable, and a consistent set of predefined covariates was included for adjustment. Covariates were selected a priori based on their theoretical relevance and previously reported associations with the outcomes. All models were estimated using ordinal logistic regression and incorporated robust standard errors clustered at the healthcare centre level to account for intraclass correlation. To account for multiple testing, we applied the Benjamini–Hochberg procedure to control the false discovery rate (FDR) at 0.05 across the primary exposure tests for each outcome. The proportional odds assumption was tested using the likelihood ratio test. None of the fitted models violated this assumption. Statistical significance was set at 0.05 and all tests were two-tailed. Analyses were performed using SPSS 25.0 (SPSS Inc., Armonk, NY: IBM Corp), Stata/MP 17.0 (StataCorp LLC, TX) and R version 4.5.1.

All analyses were conducted accounting for the clustered design, using geographical region as the clustering unit. Variance estimation was performed with cluster-robust standard errors based on a linearised variance estimator. For categorical variables, chi-squared tests of independence were computed with design-adjusted statistics (Rao & Scott adjustment). For ordinal outcomes, survey-adjusted ordinal logistic regression models were fitted, reporting odds ratios and 95% confidence intervals adjusted for the cluster effect. ANOVA tests were computed without adjustment for the clustered design, as the very limited number of clusters (*n* = 5) precluded stable variance estimation within the survey framework. Results of the unadjusted ANOVA should therefore be interpreted as exploratory.

The research team held several meetings to critically assess and discuss the relevance of each variable. Variables included in the logistic models were selected based on their theoretical and epidemiological importance, as informed by previous evidence. As part of this process, a conceptual framework was developed to provide a theoretical rationale for the relationships between variables (see Supplementary Material 2). In line with the comprehensive definition of menstrual health proposed by Hennegan et al^2^ all menstrual health-related variables were included in the analyses. For sociodemographic variables, a reflexive process was undertaken to determine which variables should be included in the logistical models. This selection was based on both existing literature and the results of bivariate analyses, which further supported decisions on variable inclusion or exclusion (see Supplementary Material 2).

### Ethics approval

Ethical approval was obtained on 11^th^ January 2023, by IDIAPJGol Research Ethics Committee (Ref 22/234-P). Informed verbal and written consent were obtained from all participants prior to their participation. Anonymity and confidentiality were ensured throughout the study. All participants gave their informed consent for the use and publication of their anonymised data.

### Reflexivity statement

The team comprises professionals with expertise in public health, psychology, primary health care, statistics, nursing and medicine. Team members are based in five regions across Spain: Catalonia, the Basque Country, Andalusia, Galicia and the Canary Islands. The majority of the team identify as women (*N* = 22), while three members identify as men. The principal investigator and corresponding author holds a PhD in Health Psychology and has extensive experience and leadership in research projects, especially in the areas of menstrual equity and menstrual health. Other team members also bring significant experience in research, with several experts in quantitative methodology. Each participating region includes researchers with a longstanding track record in research projects related to sexual and reproductive health and mental health. The team is committed to conducting public health research informed by a social determinants of health approach and a gender-based perspective.

## Results

### Sociodemographic, menstrual health, and mental health characteristics

A total of 1,404 individuals participated in the study. The mean age was 34.7 (SD = 10.1). The majority identified as women (99.4%) and as heterosexual (86.1%). Most participants were born in Spain (84.2%) and held Spanish nationality (90.9%). Nearly half had completed university education (47.8%). Regarding mental health, 41.2% of participants reported symptoms consistent with mild depression and 40.2% reported mild anxiety. In terms of menstrual health, 43.1% reported experiencing moderate menstrual pain and 22.4% reported high-intensity menstrual pain. Moderate premenstrual symptom frequency was reported by 52.3% of participants, while 15.2% reported high frequency. Most participants felt comfortable changing menstrual products primarily at home (83.7%). Moderate and high levels of menstrual poverty were reported by 14.1% and 7.3% of participants respectively. In terms of social participation, 43.2% reported moderate impairments during menstruating. See Table 1 for further details.

**Table 1.** Sociodemographic, mental health (anxiety and depression) and menstrual health characteristics (*N* = 1404)

Variable	***N* (%)**
**Sociodemographic characteristics**	
**Age** [Mean (SD*)]	34.7 (10.1)
18–25	336 (23.9)
26–35	417 (29.7)
36–45	375 (26.7)
≥46	276 (19.7)
**Gender**	
Women	1396 (99.4)
Non-binary/gender fluid	5 (0.4)
Men	3 (0.2)
**Trans**	
Yes	14 (1.0)
No	1390 (99.0)
**Sexual orientation**	
Heterosexual	1209 (86.1)
Homosexual	66 (4.7)
Bisexual	114 (8.1)
Asexual	8 (0.6)
Pansexual	7 (0.5)
**Country of birth**	
Spain	1182 (84.2)
Other	222 (15.8)
**Administrative situation**	
Spanish nationality	1276 (90.9)
Permanent residency	66 (4.7)
Temporal residency	22 (1.6)
No permit/In process	40 (2.8)
**Completed education**	
No formal education	12 (0.9)
Primary education	97 (6.9)
Secondary education	623 (44.4)
University education	672 (47.8)
**Employment status**	
Working full-time/part-time/self-employed	987 (70.3)
Studying	235 (16.7)
Unemployed (without benefits)/unpaid carer	171 (12.2)
Unemployment with benefits	89 (6.3)
**Informal caregiver**	
Yes	599 (42.7)
No	805 (57.3)
**Recognised disability**	
Yes	46 (3.3)
No	1358 (96.7)
**Public healthcare card**	
Yes	1380 (98.3)
No	24 (1.7)
**Financial problems (<12 months)**	
Always/Almost always	177 (12.6)
Frequently/Sometimes	400 (28.5)
Almost never/Never	827 (58.9)
**Experiences of discrimination**	
Gender	390 (27.8)
Racism	95 (6.8)
Sexual orientation	54 (3.8)
Age	117 (8.3)
Functional diversity	13 (0.9)
Poverty	47 (3.3)
Religion	26 (1.9)
Fatphobia	34 (2.4)
Never been discriminated against	848 (60.4)
**Self-rated health**	
Excellent	100 (7.1)
Very good	474 (33.8)
Good	632 (45.0)
Fair	185 (13.2)
Poor	13 (0.9)
**Mental health characteristics**	
**Depression (PHQ-9)**	
None	457 (32.5)
Mild	579 (41.2)
Moderate	233 (16.6)
Moderately severe	89 (6.3)
Severe	46 (3.3)
**Impact of depression on daily life**	
Not at all difficult	684 (48.8)
Somewhat difficult	589 (42.0)
Very difficult	95 (6.8)
Extremely difficult	35 (2.5)
**Anxiety (GAD7)**	
Minimal	543 (38.7)
Mild	564 (40.2)
Moderate	192 (13.7)
Severe	105 (7.5)
**Self-reported mental health diagnoses**	
Depression	117 (8.3)
Anxiety	350 (24.9)
Bipolar disorder	9 (0.6)
Obsessive-compulsive disorder	13 (0.9)
Eating disorders (e.g. anorexia, bulimia)	22 (1.6)
Substance abuse disorders	5 (0.4)
Schizophrenia or other psychotic disorders	5 (0.4)
Personality disorders (e.g. borderline personality disorder)	5 (0.4)
No mental health diagnoses	960 (68.4)
No responses	36 (2.6)
**Menstrual health characteristics**	
**Age at menarche** [Mean (SD)]	12.4 (1.7)
**Menstrual bleeding abundance**	
< 25 ml	526 (37.5)
25–80 ml	695 (49.5)
> 80 ml	183 (13.0)
**Menstrual blood clots**	
Yes, always	419 (29.8)
Yes, sometimes	722 (51.4)
No, never	263 (18.7)
**Menstrual bleeding duration**	
≤ 2 days	91 (6.5)
3–7 days	1200 (85.5)
≥ 8 days	113 (8.0)
**Menstrual cycle duration**	
≤ 21 days	290 (20.7)
22–35 days	963 (68.6)
≥ 36 days	151 (10.8)
**Intensity of menstrual pain**	
Low	485 (34.5)
Moderate	605 (43.1)
High	314 (22.4)
**Self-reported gynaecological and systemic health conditions**	
Anaemia	189 (13.5)
Iron deficiency	290 (20.7)
Uterine myomas	81 (5.8)
Endometrial polyps	27 (1.9)
Endometriosis/adenomyosis	28 (2.0)
Polycystic ovary syndrome	125 (8.9)
Premenstrual syndrome/dysphoric premenstrual disorder	90 (6.4)
Gynaecological (ovary, cervical and uterus) and breast cancers	3 (0.2)
No diagnoses	814 (58.0)
**Use of hormonal contraception**	
Yes	524 (37.3)
No	880 (62.7)
**Type of hormonal contraception methods**	
Oral contraceptive pills	316 (22.5)
Vaginal ring	40 (2.8)
Hormonal intrauterine device	68 (4.8)
Copper intrauterine device	68 (4.8)
Contraceptive patch	6 (0.4)
Contraceptive implant	17 (1.2)
Contraceptive injection	4 (0.3)
**Frequency of premenstrual symptoms**	
Low	456 (32.5)
Moderate	735 (52.3)
High	213 (15.2)
**Impact of premenstrual symptoms on daily life**	
No impact	135 (9.6)
Low	430 (30.6)
Moderate	646 (46.0)
High	193 (13.7)
**Comfort changing menstrual products**	
*At home*	
Always/Often/Frequently	1176 (83.7)
Sometimes/Rarely/Never	204 (14.5)
Non applicable	24 (1.7)
*At work*	
Always/Often/Frequently	732 (52.1)
Sometimes/Rarely/Never	560 (39.9)
Non applicable	112 (8.0)
*At public spaces*	
Always/Often/Frequently	496 (35.3)
Sometimes/Rarely/Never	853 (60.8)
Non applicable	55 (3.9)
**Access to menstrual education**	
Low	135 (9.6)
Moderate	450 (32.1)
High	819 (58.3)
**Menstruation-related experiences of taboo, stigma and discrimination**	
Low	552 (39.3)
Moderate	520 (37.0)
High	332 (23.6)
**Satisfaction with menstrual healthcare consultations (<6 months)**	
Satisfied	822 (58.6)
Not satisfied	162 (11.5)
Non applicable	420 (29.9)
**Menstrual poverty**	
Never	824 (58.7)
Low	279 (19.9)
Moderate	198 (14.1)
High	103 (7.3)
**Social participation impairment during menstruation**	
Mildly affected	407 (29.0)
Moderately affected	606 (43.2)
Significantly affected	247 (17.6)
Highly affected	144 (10.3)

*SD: Standard Deviation.

The percentages of some variables are above 100% because participants could choose multiple categories for some questions.

### Associations between menstrual health, depression and anxiety

Moderate and moderately severe depression were more common among participants with menstrual bleeding abundance of >80 ml (24.0% and 10.4%, respectively), compared to those with bleeding of <25 ml (16.0% and 5.3%, respectively) or 25–80 ml (15.1% and 6.0%, respectively). A gradient was observed between depression severity and menstrual pain intensity. Participants with moderate, moderately severe or severe depression reported higher levels of menstrual pain (24.8%, 9.9% and 4.1%, respectively), compared to those with moderate pain (17.2%, 5.6% and 4.0%, respectively) or low pain (10.5%, 4.9% and 1.9%, respectively). A similar pattern was found in the frequency of premenstrual symptoms. Participants with moderate, moderately severe and severe depression reported higher frequencies of premenstrual symptoms (25.8%, 15.5% and 12.1%, respectively), compared to those with low symptom frequency (5.5%, 0.9% and 0.7%, respectively). Differences were also evident in relation to experiences of menstrual taboo, stigma and discrimination. Participants reporting moderate or high levels of these experiences showed higher rates of depression, compared to those with low levels. A gradient was also observed between menstrual poverty and depression, with increasing levels of menstrual poverty associated with higher prevalence of depressive symptoms. Additionally, those who reported being highly affected in their social participation due to menstruation experienced higher levels of depressive symptoms. Similar trends were observed when the analyses were stratified by anxiety and menstrual health variables. Further details are available in Tables S2 and S3 of the Supplementary Material 3.

### Logistic ordinal models between menstrual health, depression, and anxiety

Higher odds of more severe depression and anxiety were found among participants with menstrual bleeding of more than 80 ml. The same pattern was observed for participants reporting menstrual blood clots in all menstrual cycles. Similarly, higher odds of more severe depression and anxiety were observed in participants with longer duration of menstrual bleeding. A clear gradient was observed regarding the intensity of menstrual pain. The higher the pain intensity, the higher the odds of more severe depression (aOR =  2.05; 95% CI = 1.82–2.31; *p* < 0.001) and anxiety (aOR =  1.82; 95% CI: 1.43–2.30; *p* < 0.001), with the odds of more severe depression being slightly higher than those for anxiety. For premenstrual symptoms, the odds of more severe depression (aOR =  14.54; 95% CI: 10.73–19.70; *p* < 0.001) and anxiety (aOR =  12.95; 95% CI: 9.10–18.44; *p* < 0.001) increased with symptom frequency. Moreover, the greater the impact of premenstrual symptoms on daily life, the higher the odds of more severe depression (aOR =  8.53; 95% CI: 5.27–13.79; *p* < 0.001) and anxiety (aOR =  11.26; 95% CI: 6.02–21.05; *p* < 0.001). Further details are provided in Table 2.

**Table 2.** Associations between menstrual health, depression and anxiety from ordinal logistic regression models (*N* = 1404)

	Depression		Anxiety	
	aOR (95% CI*)	*p* value	aOR (95% CI)	*p* value
**Menstrual abundance**				
25–80 ml	1.00	<0.001/<0.001**	1.00	<0.001/<0.001**
<25 ml	0.95 (0.81–1.11)	0.507	0.97 (0.75–1.24)	0.793
>80 ml	1.61 (1.45–1.79)	**<0**.**001**	1.67 (1.35–2.06)	**<0**.**001**
**Menstrual blood clots**				
No, never	1.00	<0.001/<0.001**	1.00	<0.001/<0.001**
Yes, sometimes	1.18 (0.90–1.55)	0.227	1.54 (1.40–1.70)	**<0**.**001**
Yes, always	1.74 (1.53–1.98)	**<0**.**001**	2.07 (1.78–2.41)	**<0**.**001**
**Menstrual bleeding duration**				
3–7 days	1.00	0.614/0.614**	1.00	0.144/0.175**
≤2 days	1.16 (0.85–1.58)	0.362	1.45 (0.98–2.16)	0.065
≥8 days	1.01 (0.92–1.11)	0.838	1.02 (0.77–1.35)	0.879
**Menstrual cycle duration**				
22–35 days	1.00	<0.001/<0.001**	1.00	<0.001/<0.001**
≤21 days	1.12 (1.03–1.23)	0.008	1.18 (1.06–1.32)	0.002
≥36 days	1.11(0.94–1.31)	0.225	1.31 (1.13–1.52)	<0.001
**Intensity of menstrual pain**				
Low	1.00	<0.001/<0.001**	1.00	<0.001/<0.001**
Moderate	1.60 (1.32–1.96)	**<0**.**001**	1.46 (1.22–1.74)	**0**.**002**
High	2.05 (1.82–2.31)	**<0**.**001**	1.82 (1.43–2.30)	**<0**.**001**
**Frequency of premenstrual symptoms**				
Low	1.00	<0.001/<0.001**	1.00	<0.001/<0.001**
Moderate	4.66 (3.67–5.93)	**<0**.**001**	4.16 (2.99–5.78)	**<0**.**001**
High	14.54 (10.73–19.70)	**<0**.**001**	12.95 (9.10–18.44)	**<0**.**001**
**Impact of premenstrual symptoms on daily life**				
No impact	1.00	<0.001/<0.001**	1.00	<0.001/<0.001**
Light	1.90 (1.30–2.77)	**0**.**001**	2.57 (1.42–4.65)	**0**.**002**
Moderate	4.50 (3.15–6.42)	**<0**.**001**	5.86 (3.58–9.57)	**<0**.**001**
High	8.53 (5.27–13.79)	**<0**.**001**	11.26 (6.02–21.05)	**<0**.**001**
Comfort changing menstrual products at work*				
Always/Often/Frequently	1.00	0.008/0.012**	1.00	0.057/0.078**
Sometimes/Rarely/Never	1.35 (1.08–1.69)	** **	1.27 (0.99–1.63)	** **
Comfort changing menstrual products at public spaces*				
Always/Often/Frequently	1.00	0.154/0.165**	1.00	0.152/0.175**
Sometimes/Rarely/Never	1.21 (0.93–1.57)		1.22 (0.93–1.59)	
Comfort changing menstrual products at home*				
Always/Often/Frequently	1.00	0.039/0.048**	1.00	0.299/0.299**
Sometimes/Rarely/Never	0.92 (0.85–1.00)		1.10 (0.92–1.32)	** **
**Access to menstrual education**				
High	1.00	0.027/0.036**	1.00	0.016/0.024**
Moderate	1.40 (1.09–1.79)	**0**.**007**	1.30 (1.00–1.70)	**0**.**050**
Low	1.35(0.93–1.95)	0.118	1.75 (1.18–2.59)	**0**.**005**
**Menstruation-related experiences of taboo, stigma and discrimination**				
Low	1.00	<0.001/<0.001**	1.00	<0.001/<0.001**
Moderate	1.67 (1.44–1.94)	**<0**.**001**	1.57 (1.25–1.96)	**<0**.**001**
High	2.23 (1.81–2.76)	**<0**.**001**	2.34 (1.91–2.87)	**<0**.**001**
**Satisfaction in menstrual healthcare consultations (<6 months)**				
Satisfied	1.00	0.059/0.068**	1.00	0.223/0.239**
Not satisfied	1.59 (0.97–2.62)	**0**.**065**	1.44 (0.95–2.17)	0.085
Non applicable	1.17 (0.80–1.72)	0.414	1.09 (0.73–1.62)	0.668
**Menstrual poverty**				
Never	1.00	<0.001/<0.001**	1.00	<0.001/<0.001**
Low	1.55 (1.41–1.70)	**0**.**001**	1.37 (1.12–1.66)	**0**.**002**
Moderate	2.11 (1.66–2.67)	**<0**.**001**	2.11 (1.65–2.70)	**<0**.**001**
High	2.50 (1.50–4.19)	**<0**.**001**	2.55 (1.68–3.87)	**<0**.**001**
**Social participation impairment during menstruation**				
Mildly affected	1.00	<0.001/<0.001**	1.00	<0.001/<0.001**
Moderately affected	2.81 (2.05–3.87)	**<0**.**001**	2.85 (2.29–3.55)	**<0**.**001**
Significantly affected	6.68 (5.62–7.93)	**<0**.**001**	5.85 (4.61–7.43)	**<0**.**001**
Highly affected	7.41 (4.9–11.19)	**<0**.**001**	6.69 (5.22–8.59)	**<0**.**001**

An independent statistical model was estimated for each variable presented. Each OR represents the cumulative odds of being in a higher category of depression or anxiety. The logistic models were adjusted for the variables age, educational level, employment status, economic issues, sexual orientation, self-perceived health and contraceptive use.

*CI =  confidence interval.

#We eliminated the category “not applicable” for logistic models.

**Adjusted *p*-values were calculated using the Benjamini–Hochberg procedure to control the false discovery rate (FDR = 0.05).

Participants who reported sometimes, rarely, or never feeling comfortable changing menstrual products at work had higher odds of more severe depression (aOR =  1.35; 95% CI: 1.08–1.69; *p* = 0.012) and anxiety (aOR =  1.27; 95% CI: 0.99–1.63; *p* = 0.078). Similarly, those who reported sometimes, rarely, or never feeling comfortable changing menstrual products in public spaces showed increased odds of more severe depression and anxiety. Participants with moderate access to menstrual education had higher odds of more severe depression (aOR =  1.40; 95% CI: 1.09–1.79; *p* = 0.007) and anxiety (aOR =  1.30; 95% CI: 1.00–1.70; *p* *=* 0.050). A gradient was observed between menstruation-related experiences of taboo, stigma and discrimination and mental health outcomes. Participants reporting higher levels of these experiences had increased odds of more severe depression (aOR =  2.23; 95% CI: 1.81–2.76; *p* < 0.001) and anxiety (aOR =  2.34; 95% CI: 1.91–2.87; *p* < 0.001). The odds of more severe depression (aOR =  2.50; 95% CI: 1.65–3.78; *p* < 0.001) and anxiety (aOR =  2.55; 95% CI: 1.70–3.83; *p* < 0.001) increased as experiences of menstrual poverty were higher. A gradient was also observed between the impact of menstruation on social participation and both depression and anxiety. The greater the impact on social participation, the higher the odds of more severe depression (aOR =  7.41; 95% CI: 4.90–11.19; *p* < 0.001) and anxiety (aOR =  6.69; 95% CI: 5.22–8.59; *p* < 0.001). See Table 2 for further information.

The logistic models were stratified by sociodemographic characteristics. The odds of more severe depression were higher among participants who reported moderate or high menstrual pain and were not born in Spain, compared to participants who were born in Spain. Regarding social participation during menstruation, there were increased odds of depression across all age groups, with the strongest association in participants aged 36–45 years (aOR = 16.50; 95% CI: 9.48–28.70; *p* < 0.001). When stratified by education level, the odds of more severe depression were higher among participants with higher reports of menstrual poverty who had completed only primary education (aOR =  4.65; 95% CI: 1.87–11.54; *p* = 0.001), compared to those with secondary education (aOR =  2.83; 95% CI: 1.48–5.42; *p* = 0.002) and university education (aOR =  2.34; 95% CI: 0.78–7.03; *p* = 0.131). A similar pattern was observed when stratifying by financial constraints. Moderate menstrual poverty combined with reporting financial issues always or almost always increased the odds of more severe depression (aOR =  3.47; 95% CI: 1.14–10.53; *p* = 0.028), compared to those reporting financial issues frequently, sometimes, almost never or never.

The odds of more severe anxiety were higher among participants with heavy menstrual bleeding (>80 ml) and born outside of Spain, compared to those born in Spain. When stratifying by age, higher odds of more severe anxiety were observed across all age groups among participants reporting moderate premenstrual symptom frequency, with the strongest association in those aged 36–45 years (aOR =  22.21; 95% CI: 9.14–53.98; *p* < 0.001). Furthermore, participants aged 36–45 who reported a high impact on social participation during menstruation had higher odds of more severe anxiety (aOR =  14.29; 95% CI: 6.13–33.533; *p* < 0.001), compared to other age groups. When stratifying by sexual orientation, the odds of more severe anxiety were higher among participants reporting high menstrual poverty who identified as non-heterosexual (aOR =  5.43; 95% CI: 1.98–14.86; *p* = 0.001), compared to heterosexual participants (aOR =  2.24; 95% CI: 1.26–3.99; *p* = 0.006).

## Discussion

This study demonstrates an association between depression and anxiety and various menstrual health outcomes, such as menstrual abundance, presence of blood clots, intensity of menstrual pain, frequency and impact of premenstrual symptoms, comfort in changing menstrual products at work, experiences of menstrual-related taboo, stigma and discrimination, menstrual poverty, access to menstrual education, and impaired social participation during menstruation. The observed patterns in the associations between menstrual health and depression, and those between menstrual health and anxiety were highly similar. This may be attributed to the frequent co-morbidity between depression and anxiety, and the overlap in their symptomatology (e.g. rumination).^28^

Menstrual bleeding exceeding 80 ml and reports of menstrual blood clots were associated with both depression and anxiety in our study. Scientific evidence supports the association between heavy menstrual bleeding and both depression and anxiety,^29,30^ with some studies linking this to hormonal changes in the hypothalamic–pituitary–adrenal (HPA) axis.^31^ Chronic stress can dysregulate the HPA axis, disputing the release of luteinising hormone and progesterone, both of which play a pivotal role in regulating the menstrual cycle.^31^ Moreover, sustained stress adversely effects mental health, as HPA axis hyper-activation and elevated cortisol levels are associated with the onset and worsening of depression and anxiety. Menstrual blood clots and heavy menstrual bleeding are also associated with iron deficiency and anaemia, which can contribute to symptoms such as increased fatigue, anxiety and dizziness.^32^

Our findings suggest that menstrual pain tends to increase alongside the severity of depression and anxiety,^33^ as well as the significant emotional consequences resulting from the interference of menstrual pain with daily activities and quality of life (e.g. school or work absenteeism/presentism, and limited social interactions).^34^ The systemic neglect and normalisation of menstrual pain at both social and institutional levels (e.g. in healthcare settings) is strongly associated with greater mental and emotional health impacts among women and PWM.^35^ The socialisation of women into pain involves the acceptance of pain as an inevitable aspect of their lived experience.^14^ This may have contributed to the perpetuation of narratives that position menstrual pain as “normal” or “expected”. Such normalisation not only delegitimises and minimises women's experiences, but also hinders the search for appropriate responses and exacerbates the emotional distress of women and PWM.^14^ Moreover, it is crucial to consider how androcentrism in the health sciences has historically neglected and minimised the expression of women's discomfort (e.g. menstrual pain).^7^ As previously proposed, the dysregulation of the HPA axis may be associated with the experience of menstrual pain, depression and anxiety. However, biomedical explanations have often attributed endocrine differences between men and women to the menstrual cycle, and suggested that such variation underlies women’s greater susceptibility to mental health conditions. This has contributed to psychiatrisation and medicalisation^14^ of the emotional impacts of menstrual pain and other forms of discomfort.

The association between an increased frequency of premenstrual symptoms and poorer mental health outcomes was reported both in our study and in previous literature.^29^ It is also noteworthy to highlight the paucity of research evaluating associations between premenstrual symptoms, depression and anxiety, as most studies have focused on “premenstrual syndrome” rather than “premenstrual symptoms”. The causal pathways underlying premenstrual symptoms are multifactorial. The HPA axis is involved in triggering premenstrual symptoms.^36^ As previously noted, certain social discourses have positioned women (and their discomforts) as inherently pathological, linking (pre)menstrual experiences to “hysteria” and “madness”.^11,15^ The assumption that the bodies of women and PWM are pathological and prone to irrational emotions may have contributed to the systemic underestimation, minimisation and invalidation of (pre)menstrual experiences, as well as the psychiatrisation of (pre)menstrual symptoms.

As evidenced by our findings and those of previous studies in other contexts, a lack of access to information about menstruation is associated with increased anxiety.^37^ Previous research, primarily conducted with adolescent populations at menarche, has established a correlation between inadequate menstruation-related information and adverse emotional outcomes, such as fear.^38^ Some interventions have proven effective in reducing anxiety and menstrual concerns among adolescents,^39^ highlighting the importance of ensuring access to menstrual information and education. Future studies should explore the relationship between access to menstrual information and specific mental health outcomes in adult women and PWM.

The results of this study, along with previous literature,^8,40^ highlight that both depression and anxiety are associated with menstrual poverty in adult women.^6,9^ Our findings also suggest a strong link between socioeconomic deprivation and menstrual poverty. This is consistent with previous evidence highlighting that mental health tends to deteriorate under worsening living conditions, housing insecurity,^41^ lower educational attainment, unemployment, lack of social support,^42^ and among women and PWM in manual social classes. Poverty is also a gendered phenomenon.^43^ This “feminisation of poverty” highlights that women and PWM are more likely to experience poverty due to systemic gender inequities. The impact of (menstrual) poverty on women and PWM extends to their mental health, which is profoundly shaped by gender-based expectations and norms embedded in patriarchal structures.^43^ These gendered constructs may lead to the pathologisation of women’s emotional responses and further stigmatise their mental health concerns.^14^ For instance, societal norms may dismiss women's mental health struggles as “emotional instability”, thereby failing to recognise the real impact of socioeconomic deprivation and gender inequities. Intersectional research that analyses the interplay between different social determinants of health (e.g. socioeconomic status, gender) is necessary to comprehensively understand the lived experiences of menstrual poverty.

This study suggests that individuals who feel uncomfortable changing menstrual products at work are more likely to report symptoms of depression and anxiety, whereas this association was not observed in private spaces, contrary to previous evidence.^44^ Lack of access to adequate menstrual management facilities has been associated with feelings of discomfort and insecurity,^45^ and can result in heightened anxiety and stress.^46^ This can be explained by the social imperative to conceal menstruation and relegate it to the private sphere, as menstruation is not considered a “hegemonic experience”. Such norms reflect forms of social control and foster self-policing practices that marginalise women and PWM, rendering their common bodily experiences invisible.^16^

Our results indicated a relationship between anxiety, depression and experiences of menstrual taboo, stigma, and discrimination among women and PWM. Previous research has similarly highlighted the emotional consequences of menstrual taboo, stigma and discrimination (e.g. low self-esteem, chronic stress).^8,37^ Furthermore, the ongoing emotional toll of navigating menstrual taboos, stigma and discrimination can be significant and, at times, overwhelming.^7^ It is essential to recognise how social and cultural contexts not only generate and perpetuate menstrual taboo, stigma and discrimination, but also reinforce their negative impact on the emotional and mental health of women and PWM. These structures contribute to an environment in which menstruation is marginalised, thereby compounding feelings of shame, isolation and psychological distress.

The previously discussed issues are closely related to another finding of the study: social impairments during menstruation were associated with higher levels of anxiety and depression. In particular, women aged 35–46 who reported considerable social impairments while menstruating were the most affected by both anxiety and depression. This life stage often involves increased responsibilities in both reproductive and productive spheres, such as caring for children or elders, as well as heightened job-related demands. The so-called “crisis of care”,^47^ along with the cumulative burden of managing multiple roles on a daily basis, may help explain the observed association with anxiety and depression. Besides, community-based support networks may play a protective role in mitigating the risk of poor mental health.

Menstrual and mental health must be addressed from a gender- and feminist-informed perspective and understood in relation to other social determinants of health (e.g. age, race, social class or sexual orientation). Our findings suggest that the relationship between menstrual health outcomes and mental health may be mediated by sociodemographic context. For instance, individuals not born in Spain reported worse mental health outcomes in relation to specific menstrual health variables, such as menstrual pain and menstrual poverty. Migratory processes, along with interpersonal and institutional racism, may help explain these associations, as previous research has shown that these experiences of violence are intrinsically linked to poorer mental health outcomes.^48^ These stressors may also influence menstrual characteristics, such as irregular cycles or increased menstrual pain. Therefore, applying an intersectional lens to menstrual health research is crucial for advancing our understanding of how social structures of power and oppression produce and sustain menstrual and mental health inequities. As highlighted in previous critical menstrual studies, these complex and deeply embedded structures must remain central to critical menstrual research.

### Strengths, limitations and reflexive accounts

The main strength of this study is that it provides novel data on menstrual health^2^ and self-reported experiences of depression and anxiety in women and PWM in Spain. Another strength lies in the composition of a broad, multidisciplinary research team, which included academics from various disciplines as well as healthcare professionals. Additional strengths related to the recruitment process included the use of a hybrid survey format (online and paper-based), and the relationships of trust established between recruiters and participants. Moreover, the topic of the survey itself facilitated participation by fostering self-awareness around menstrual and mental health and by helping to mitigate the perceived waiting time before healthcare consultations.

We acknowledge several limitations in our study. First, its retrospective design does not allow for the determination of causal relationships. However, it offers an initial approximation toward a comprehensive understanding of the association between menstrual health, anxiety and depression in our context. Our analysis may carry an increased risk of type I error (e.g. false-positive findings) due to multiple testing. Nevertheless, given the exploratory nature of the study, it was deemed appropriate to present a broad overview of conceptually relevant associations, including those that did not reach statistical significance. The use of a non-probabilistic sampling method limits the generalisability of our findings. As participants were not randomly selected, the sample may not accurately represent the broader population, and results may only be applicable to population groups with characteristics similar to those of our study sample. Self-reported variables may also be subjected to recall bias, particularly those covering a six-month recall period. Despite efforts to include hard-to-reach populations, some groups of women and PWM are underrepresented in our sample. Consequently, findings should be interpreted with caution when applied to hard-to-reach populations. The 20-minute duration of the survey contributed to refusals and incomplete questionnaires. Additionally, some participants found it challenging to complete the survey on a mobile device or to fully understand certain questions. Other challenges encountered during the recruitment process included heavy workloads of healthcare professionals and the dissatisfaction expressed by some users with primary healthcare services. Furthermore, due to the anonymous nature of the questionnaire, the research team was unable to identify participants based on their responses to the anxiety and depression scales, and therefore could not provide direct clinical referrals. However, as the recruiters were primary healthcare professionals, it is possible that some of the identified mental health concerns were already being addressed in the course of routine clinical practice.

Some limitations related to the questionnaire should also be noted. Only a few scales (mainly PHQ-9 and GAD-7) had been previously validated, due to a lack of validated instruments on menstrual heath. To address this gap, the research team developed questions based on existing literature and previous research. While this approach may be seen as a limitation, it also represents an opportunity for future research to validate and refine these measures across diverse contexts. Moreover, validated mental health instruments may obscure the complexity of emotional and psychological experiences of women and PWM. This may reflect on how the development of such instruments has been shaped by biomedical hegemony and patriarchal conceptualisations of mental health. Another limitation of the present study is the limited depth of exploration of complex concepts, such as menstrual stigma and discrimination. However, the primary objective was to provide a comprehensive overview of the multifaceted components that constitute menstrual health, laying the groundwork for more in-depth and qualitative studies in the future. Another limitation of our study is that menstrual bleeding was assessed using self-reported volume categories, without capturing the functional impact on participants’ daily lives. This method may lead to overestimation, in which menstruators report high perceived bleeding without meaningful disruption, or underestimation, in which lower-volume bleeding substantially affects quality of life. Validated instruments, such as the SAMANTA scale,^49^ specifically measure the functional consequences of menstrual bleeding, offering a more accurate assessment. Future studies could benefit from integrating these tools alongside self-reported volume estimates. Nonetheless, our use of standardised absorbency-based categories is aligned with prior research and clinical guidelines, ensuring comparability across studies.^50–52^

### Implications for research, policy and practice

This study has several important implications. Healthcare professionals must be informed about the close interrelationship between menstrual health and mental health, as well as other key determinants (e.g. socioeconomic status, age) that influence the menstrual health of women and PWM. Given that a hegemonic biomedical perspective often reduces healthcare responses to medicalisation and normalisation of (pre)menstrual experiences,^17^ it is essential to promote comprehensive training for healthcare professionals. Such training should incorporate a critical understanding of social inequities in both menstrual and mental health and move beyond narrow, diagnosis-focused conceptualisations of mental health. Instead, it should address emotional well-being and discomforts across a broad spectrum and explore how these interact with the menstrual cycle. A person-centred approach is particularly relevant to account for the complex dynamic interrelations between menstrual and mental health. This perspective should not be limited to healthcare services but extended to educational systems (e.g. menstrual health education in schools, inclusive to all genders) and community-based support structures, and policymaking processes. In 2023, Spain passed its first legislation on menstrual health (Ley Orgánica 1/2023), which includes rights related to the workplace, access to menstrual products, menstrual education with an intersectional and human rights approach, and menstrual health research.^1,53^ While this is a significant step forward, there remains an urgent need to develop a more comprehensive legal and policy framework that also addresses emotional and mental health in connection with menstrual health. The findings of this study can contribute to the development of future legislation and policies in our context, by highlighting the intersections between menstrual and mental health. Additionally, future research could explore the causal pathways between menstrual health, depression and anxiety. Longitudinal designs could be especially useful to examine these relationships over time and to better understand the underlying mechanisms. Further research is also needed to focus on population groups disproportionately affected by poor menstrual and mental health, and to develop validated menstrual health indices that reflect its multidimensional nature. Such tools could improve the operationalisation, analysis and cross-contextual comparison of menstrual health data.

## Conclusion

This study shows associations between poorer menstrual health outcomes and depression and anxiety. Socio-economic factors, such as age, financial constraints and country of birth, were found to mediate the relationships between menstrual health and depression and anxiety. These findings contribute to a deeper understanding of how menstrual and mental health are intertwined, through the lens of gender and social health inequities. Our research may support evidence-based informed community interventions and policymaking in the Spanish context. Training for healthcare professionals must be strengthened, along with the allocation of greater resources to health services. It is essential to move beyond narrow definitions of mental health that focus solely on clinical diagnoses, and to consider the full spectrum of emotional health and discomforts, particularly in relation to the menstrual cycle. This broader approach could help align menstrual healthcare more closely with the actual needs of women and PWM. Importantly, these findings also call for action beyond the healthcare system. Educational and community sectors should incorporate menstrual education and promote community-based initiatives that recognise the interconnection between menstrual and mental health. Menstrual interventions, policies and research must adopt a gender-sensitive and social determinants of health perspective, moving away from the pathologisation of menstrual experiences and discomforts. At the same time, structural determinants of anxiety and depression in women and PWM must be acknowledged and addressed.

## Supplementary Material

Figure S1. Conceptual framework depicting theoretical associations between “menstrual health”, “mental health”, and “sociodemographic characteristics” variables.

Table S1. STROBE Statement–checklist of items that should be included in reports of observational studies

## Data Availability

The datasets generated and analysed during the current study are available from the corresponding author on reasonable request. Datasets are not publicly available to maintain participants’ anonymity and confidentiality. Data are stored securely in [name of institution] servers. Anonymity and confidentiality were ensured using an anonymous online questionnaire that did not collect participants’ identifiable data (e.g. names, email addresses). The research team responsible for processing the data had no direct contact with the participants. Recruiters had no access to the data provided by the participants.

## Footnotes

* We refer to the systemic conditions (e.g. economic, social, cultural) that shape women’s and PWM’s lives.

† Bio-medicalisation is understood as a phenomenon whereby biomedical knowledge and technologies intervene in an increasing number of aspects of life, expanding their scope from illness to health, and to the management of bodies and lives. (17)

‡ Androcentrism refers to a perspective in which male experiences are taken as the norm, often marginalising or making invisible the experiences of women and other gender-diverse people.

§ Menstrual bleeding was assessed via participant self-report using standardised categories based on absorbency proxies, including the number of regular tampons, pads, or equivalent menstrual cup volume. The categories were defined as <25 ml, 25–80 ml and >80 ml per period. These thresholds align with commonly used clinical definitions of heavy menstrual bleeding and research thresholds associated with relevant health outcomes. For example, the FIGO systems and classification for normal and abnormal uterine bleeding.^50^

** This variable refers to a group of health conditions, both general and specific to the reproductive system, that may be associated with menstrual health. This includes, for example, anaemia, iron deficiency, uterine fibroids, endometrial polyps, endometriosis/adenomyosis, polycystic ovary syndrome, premenstrual syndrome or premenstrual dysphoric disorder, and gynaecological cancers (e.g., uterine or ovarian), among others.

†† The selected items were: (1) “My period has forced me to slow down my usual pace of life”; (2) “Pain has interfered with my work, academic or domestic activities”; (3) “Menstrual bleeding has affected my work, academic or domestic activities”; (4) “Fatigue has forced me to decrease the intensity of what I am doing”; (5) “My mood has interfered with my work, academic or domestic activities”; (6) “My performance in work or academic activities has been affected”; (7) “Pain has affected my social and leisure activities”; (8) “Menstrual bleeding has affected my social and leisure activities” and (9) “Fatigue has limited my social and leisure activities”. The remaining 13 items from the scale were not included in the construction of the social participation variable (e.g. “I have experienced nausea and/or vomiting”).
