# Supplementary material for: Menstrual health outcomes and associations with depression and anxiety in Spain: a cross-sectional study
Source: Sex Reprod Health Matters. 2026 Jul 31;33(1):2702119. doi: 10.1080/26410397.2026.2702119 (PMC13700537; doi:10.1080/26410397.2026.2702119)
Supplement: Supplementary file 2 [file ZRHM_A_2702119_SM1539.docx]

**Figure S1.** Conceptual framework depicting theoretical associations between “menstrual health”, “mental health”, and “sociodemographic characteristics” variables.

*In order to facilitate understanding of the multiple relationships between the variables, the associations between menstrual health and sociodemographic characteristics are represented with numbers, while those between menstrual health and mental health are represented with letters. Relationships between mental health and sociodemographic characteristics are shown with symbols. Each number, letter, and symbol in the figure is intended to represent a theoretically plausible relationship between two variables, based on the evidence.*

Other considerations:

The selection of variables for the construction of the logistic models was carried out through a reflective and informed process, considering both previous evidence and the results of the bivariate analyses in this study. Despite the demonstrated associations between menstrual health and multiple sociodemographic variables, those with the strongest theoretical support were prioritised as adjustment variables in the logistic models. Sociodemographic variables included: age, completed education, employment status, financial problems, and self-rated health. Sexual orientation was also included in the study, although its correlation with menstrual and mental health has not been extensively addressed in the literature. Contraceptive use was also included as an adjustment variable in the logistical models, given its association with menstrual and mental health.

Other variables were not incorporated into the logistic models due to limitations in the sample size for specific variable categories, such as gender, identifying as trans, experiences of discrimination, having access to public healthcare, and having a recognised disability. Although variables such as country of birth, administrative status and acting as an informal caregiver are of clear conceptual relevance, they were excluded from the final models to minimize the risk of overadjustement and preserve model parsimony and statistically stability.
